# Differences in Visceral Fat and Fat Bacterial Colonization between Ulcerative Colitis and Crohn’s Disease. An *In Vivo* and *In Vitro* Study

**DOI:** 10.1371/journal.pone.0078495

**Published:** 2013-10-24

**Authors:** Alessandra Zulian, Raffaella Cancello, Chiara Ruocco, Davide Gentilini, Anna Maria Di Blasio, Piergiorgio Danelli, Giancarlo Micheletto, Elisabetta Cesana, Cecilia Invitti

**Affiliations:** 1 Diabetes Research Laboratory, Istituto Auxologico Italiano, Milan, Italy; 2 Laboratory of Molecular Biology, Istituto Auxologico Italiano, Milan, Italy; 3 Department of Clinical Sciences, Luigi Sacco Hospital, University of Milan, Milan, Italy; 4 Department of Medical and Surgical Pathophysiology and Transplants, University of Milan, Milan, Italy; 5 Microbiology Laboratory, Istituto Auxologico Italiano, Milan, Italy; 6 Department of Medical Sciences and Rehabilitation, Istituto Auxologico Italiano, Milan, Italy; Charité, Campus Benjamin Franklin, Germany

## Abstract

Crohn’s disease (CD) is notably characterized by the expansion of visceral fat with small adipocytes expressing a high proportion of anti-inflammatory genes. Conversely, visceral fat depots in ulcerative colitis (UC) patients have never been characterized. Our study aims were a) to compare adipocyte morphology and gene expression profile and bacterial translocation in omental (OM) and mesenteric (MES) adipose tissue of patients with UC and CD, and b) to investigate the effect of bacterial infection on adipocyte proliferation *in vitro*. Specimens of OM and MES were collected from 11 UC and 11 CD patients, processed and examined by light microscopy. Gene expression profiles were evaluated in adipocytes isolated from visceral adipose tissue using microarray and RTqPCR validations. Bacteria within adipose tissue were immuno-detected by confocal scanning laser microscopy. Adipocytes were incubated with *Enterococcus faecalis* and cells counted after 24h. Morphology and molecular profile of OM and MES revealed that UC adipose tissue is less inflamed than CD adipose tissue. Genes linked to inflammation, bacterial response, chemotaxis and angiogenesis were down-regulated in adipocytes from UC compared to CD, whereas genes related to metallothioneins, apoptosis pathways and growth factor binding were up-regulated. A dense perinuclear positivity for *Enterococcus faecalis* was detected in visceral adipocytes from CD, whereas positivity was weak in UC. *In vitro* bacterial infection was associated with a five-fold increase in the proliferation rate of OM preadipocytes. Compared to UC, visceral adipose tissue from CD is more inflamed and more colonized by intestinal bacteria, which increase adipocyte proliferation. The influence of bacteria stored within adipocytes on the clinical course of IBD warrants further investigations.

## Introduction

The two major forms of chronic inflammatory bowel disease (IBD), ulcerative colitis (UC) and Crohn’s disease (CD), are believed to result from interactions between the environment, genetic predisposition, unbalanced host-commensal microbiota and a widespread immune defect [1]. However, UC and CD present distinct pathogenic mechanisms and characteristics [1], one being the expansion of mesenteric fat surrounding inflamed intestinal tracts (“creeping fat”), which is typical for CD and absent in UC. 

We have recently demonstrated that, in patients with active CD, omental (OM) visceral adipose tissue shows the same inflammatory morphology and molecular profile of creeping fat (MES) [2]. In addition, we reported that OM adipocytes from CD patients are smaller than those of normal-weight subjects and express a higher proportion of anti-inflammatory genes compared to obese patients. These findings suggest that the adipocyte undergoes beneficial changes [3] and we hypothesized that the intra-abdominal fat expansion of CD is a protective phenomenon aimed at controlling the inflammatory response and preventing dissemination of intestinal bacteria. 

It is worth recalling that gut microbiota and/or their products have been shown to promote inflammation and determine the anti-inflammatory response of visceral adipose tissue in obese patients [4]. Further, bacterial translocation to MES has been demonstrated in mice with experimental UC-like colitis and in humans with CD [5]. Conversely, morphology and molecular profiles of visceral fat in UC as well as the effects of bacterial translocation on adipocytes from IBD patients have yet to be characterized.

Given that intestinal bacteria translocation into intra-abdominal fat depots of IBD patients may affect both adipocyte morphology and gene expression, we investigated visceral fat depots and bacterial translocation in OM and MES from UC and CD patients. In addition, we analyzed the effects of intestinal bacteria on visceral adipocyte proliferation *in vitro*. 

## Materials and Methods

### Patients and specimen collection

Eleven patients with UC (3 severe pancolitis, 3 mild pancolitis, 2 proctitis and 3 left-sided colitis), and 11 patients with active CD (7 ileo-colic, 1 colonic and 3 ileal) were recruited from those referred to surgery for complications. Three UC patients were hypertensive and two had type 2 diabetes. None of the CD patients had hypertension and/or diabetes. All CD and UC patients had discontinued corticosteroids, immuno-modulators, salicilates or infliximab since at least 2 months. 

A fasting blood sample was collected the day before surgery for measurement of glucose, HDL- and LDL-cholesterol, triglycerides, fibrinogen and C-reactive protein in all patients. 

Adipose tissue specimens were taken from OM and MES close to the affected intestine. Major efforts were made to guarantee sterility during tissue collection. Each adipose tissue specimen was a) fixed in 4% phosphate-buffered formalin for 24h at 4°C, then processed for haematoxylin/eosin and immunostaining; b) digested with 1 mg/ml collagenase type II (Sigma, St. Louis, US) for isolation of mature adipocytes for gene expression arrays and bacterial infection experiments (for details see below).

The Ethical Committee of the Istituto Auxologico Italiano approved the study protocol. Written informed consent was obtained from all patients prior to sample collection.

### Biochemical measurements

Serum levels of glucose and lipids were measured using an automated analyzer (Roche Diagnostics, Mannheim, Germany). Fibrinogen was measured on citrate plasma with a clot-rate assay (ACL 200/IL, Instrumentation Laboratory, Milan, Italy). The sensitivity of this assay is 7.5 mg/dl and the intra- and inter-assay CVs are 4.8 and 5.2%, respectively. C-reactive protein was measured by an immuno-turbidimetric assay (CRP Latex HS, Roche Diagnostics, Mannheim, Germany); sensitivity is 0.03 mg/l and intra- and inter-assay CVs are 1.3 and 5.7%, respectively.

### Morphologic analysis

Adipose tissue samples were formalin-fixed overnight at 4°C, washed in 1X phosphate buffer saline (PBS) and paraffin embedded. Sections were cut at 5 µm thickness and then stained with haematoxylin and eosin or used for bacterial immunostaining (see later). Adipocyte cell diameter was assessed by light microscopy on 5 randomly selected sectional areas (10X, Leica DMR, Wetzlar, Germany) and at least 200 cells were measured using an image analysis system (Leica QWin image analysis and processing).

### Isolation of human adipocytes

Immediately after removal, a fragment of adipose tissue was cut into small pieces and digested with 1 mg/ml collagenase type II (Sigma, St. Louis, US) for at least 1 hour at 37°C with horizontal agitation. Digested tissue was filtered first through sterile gauze and then through 100 µm nylon filter (BD Bioscience 1 Becton Drive Franklin Lakes, New Jersey, USA). Filtrates were centrifuged at 500xg and floating adipocytes washed in PBS, collected and stored in TRIzol (Invitrogen, Carlsbad, CA, USA) until RNA extraction.

The purity of adipocytes was verified using RNA markers of macrophage [(*MCP-1, CD206* and *CD163*)] and adipocyte [fatty acid binding protein (*FABP4*), perilipin (*PLIN*), leptin (*LEP*), peroxisome proliferator-activated receptor gamma (PPARG)] cells.

### RNA preparation and cDNA synthesis

The RNA from isolated adipocytes was extracted using TRIzol plus RNA Purification Kit (Invitrogen Corporation, Jefferson City, US) as per manufacturer’s instructions. RNA concentrations were quantified by spectrophotometer and RNA integrity verified by agarose gel electrophoresis.

### Microarray analysis and functional annotations

Gene expression profiles were analyzed using the Human HT-12 v3 BeadChips whole-genome gene expression direct hybridization assay (Illumina, San Diego, CA), as previously described [2]. Microarray experiments were performed on duplicates of 4 different pools of RNAs extracted from adipocytes isolated from OM and MES of UC patients (n=5) and CD patients (n=5), respectively (NCBI’s GEO repository, accession number GSE46754).

Prioritized differentially expressed gene lists obtained by Genome Studio software (Illumina) were uploaded onto DAVID (Database for Annotation, Visualization, and Integrated Discovery) [6] and annotated automatically. Microarray data were MIAME compliant [7].

### Validations of microarray data by Real Time quantitative PCR (RTqPCR)

Copy DNAs obtained by reverse-transcription with SuperScript III (Invitrogen, Carlsbad, CA) from 500 ng total RNA were used for quantitative gene expression. The following Assay-on-Demand probes (Applied Biosystems by Life Technologies Italia, Monza, Italy) were used: interleukin 6 (IL6), tumor necrosis factor alpha (TNFA), monocyte chemoattractant protein 1 (MCP1), metallothionein 1G (*MT1G*) and 1E (*MT1E*), lipopolysaccharide-binding protein (LBP) and defensin beta 1 (*DEFB1*). Human ribosomal protein LP0 (*RPLP0*) was used as housekeeping gene. The data were analysed using the SDS v.3 software (Software Diversified Systems, Spring Lake Park, MN). Quantification of unknown samples was performed by 2^deltadeltaCt (i.e., target gene mRNA is normalized to *RPLP0* expression by subtracting the Ct for the housekeeping gene from the Ct for the gene of interest) and expressed as arbitrary units (AU).

### *In vitro* effect of bacterial infection on adipocyte proliferation

Fragments of OM collected from 2 UC and 2 CD patients were digested with 1 mg/ml collagenase type II (Sigma, St. Louis, US) as previously described [2]. Stromal vascular fraction cells (SVF) were isolated by centrifugation and cultured in 1:1 Ham’s F12/DMEM (Invitrogen Corporation, Jefferson City, US) supplemented with 10% decomplemented Fetal Bovine Serum (FBS) (Sigma, St. Louis, US), penicillin, streptomicin and amphotericin B. At sub-confluence, cells were starved for 3h in Ham’s F12/DMEM 1%FBS without antibiotics and then infected for 24h with 0.001 McFarland (=100.000 CFU/ml) *Enterococcus faecalis* (Lyfocults, Biomerieux Inc., Durham, NC) in the latter medium. *Enterococcus faecalis* was chosen because it is a common cause of systemic infection and lethality in humans [8]; length of incubation and bacterial concentration allowing greater cell survival and viability were chosen. *In vitro* infections with *Escherichia coli* and *Staphylococcus aureus* were not suitable due to the increased adipose cell death.

After 24h infection with *Enterococcus faecalis*, cells were repeatedly washed with PBS and counted on Beckmann cell counter (Z2 coulter counter, Beckman Coulter, Inc.). Infection experiments were also performed on *in vitro* differentiated adipocytes, i.e. after 10-day differentiation with Stempro adipogenesis differentiation kit (Life Technologies Italia, Monza, Italy). After bacterial infection, intracellular triglyceride storage levels were assessed by AdipoRed staining, according to the manufacturer’s procedure (Lonza, Milan, Italy).

### Confocal microscopy

Sections (5 µm) of MES and OM from UC and CD patients were deparaffinised and hydrated, permeabilized for 10 minutes with PBS containing 0.3% triton X-100 (PBST) and then incubated for 30 minutes with 1% Bovine Serum Albumin (BSA) in PBST at room temperature (RT). Sections were then incubated for 1h at RT with a polyclonal antibody raised against *Enterococcus faecalis* (1:500 dilution, Ab-cam, Cambridge, UK) in 1% BSA-PBST. Slides were washed thrice in PBST and incubated for 30 minutes at RT with a secondary antibody (1:200, Alexa Fluor 568 goat anti-rabbit antibody coupled with ThRed, Life Technologies Italia, Monza, Italy). After three washes in PBST, slides were mounted in DAPI-containing mounting medium (Ab-cam Cambridge, UK) and visualized on a Nikon Eclipse Ti inverted confocal microscope system (Nikon Instruments Europe B.V., Amsterdam, NL). Images were captured and analysed by Nis Elements software (Nikon Instruments Europe B.V., Amsterdam, NL). 

### Statistical analyses

The “Detection Score”, i.e. Z-value of a gene relative to Z-value of negative controls, was used to determine expression levels using Illumina Genome Studio software. Genes with detection P-value <0.05 were considered to be expressed. Illumina data was normalized using a cubic spline function. Genes with a nominal P-value ≤0.05 were considered statistically significant. Spearman’s correlation was used to evaluate relationships between average signal detection (AVG) of selected genes in microarray studies and mRNA expression levels at RTqPCR.

Differences between groups were calculated using Student’s *t* test or Mann-Whitney’s test, as appropriate. Analyses were performed using Graphpad Prism software and SPSS 19 software. P-values <0.05 were considered statistically significant.

## Results

### Patient characteristics

Clinical characteristics of patients with IBD are shown in Table 1. Compared to CD patients, UC patients were older, presented higher levels of HDL cholesterol and lower levels of triglycerides and inflammatory markers. 

**Table 1:** Clinical and biochemical characteristics of patients with UC and CD.

	**UC n=11**	**CD n=11**
**Male/Female**	6/5	5/6
**Age, years**	51.8±14.3^*^	41.0±10.6
**BMI, kg/m^2^**	23.6±3.9	21.9±3.7
**Fasting glucose, mg/dl**	96.4±43.1	93.0±17.8
**Waist circumference, cm**	78.7±10.7	79.4±13.1
**HDL cholesterol, mg/dl**	45.1±15.2 ^*^	33.3±7.0
**LDL cholesterol, mg/dl**	79.1±26.1	76.4±19.1
**Triglycerides, mg/dl**	95.2±41.1	123.4±36.2
**Fibrinogen, mg/dl**	378.0±61.7 ^*^	498.4±99.6
**CRP, mg/dl**	1.6±1.3	3.1±4.2

* P<0.05 *vs*. CD. Data are expressed as mean ± SD.

### Morphological and morphometric adipocyte characterization

**Figure 1:**
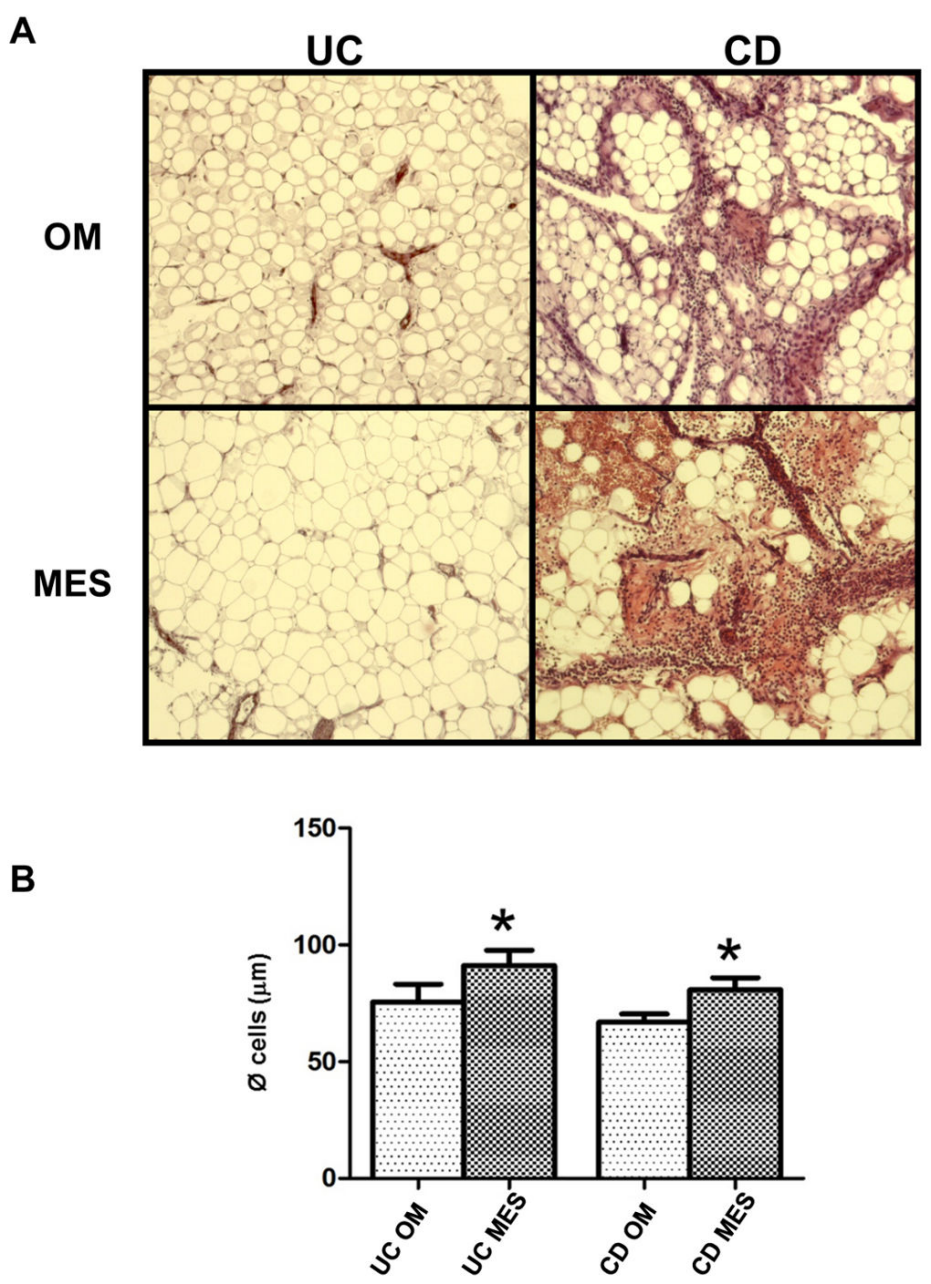
Morphological and morphometric characterization of adipose tissue from UC and CD patients. **A**. Haematoxylin-eosin staining of omental (OM) and mesenteric adipose (MES) tissue sections from one representative ulcerative colitis (UC) and Crohn's disease (CD) patient. Magnification is 10X. **B**. Mean adipocyte size from different adipose tissue depots from 11 UC and 11 CD patients. Data are expressed as means ± SE, *P<0.05 between OM and MES in UC and CD patients.

OM and MES samples were observed at light microscopy. Figure 1A shows the morphology of OM and MES compartments in one representative UC and CD patient, respectively. Both fat depots appeared to be less inflamed in UC than in CD. In UC, OM was more inflamed and fibrotic than MES. OM adipocytes were significantly smaller than those in MES from both IBD patients (Figure 1B). 

### Molecular signature of adipocytes isolated from OM and MES

**Figure 2:**
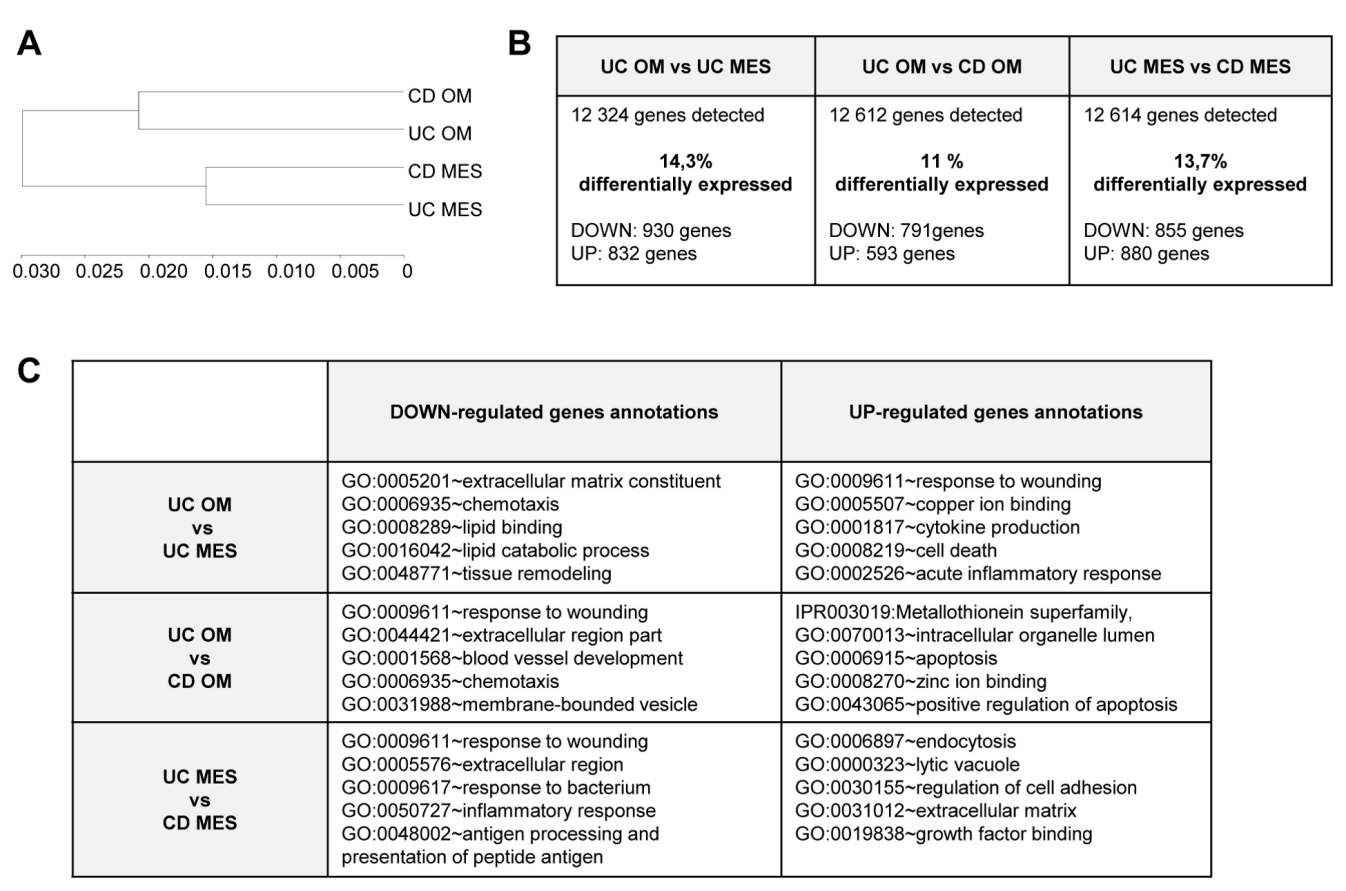
Gene expression analysis of isolated adipocytes from UC and CD patients. **A**. Clustering of global gene expression in omental adipose tissue (OM) and mesenteric adipose tissue around the involved intestinal tract (MES) in ulcerative colitis (UC) and Crohn's disease (CD) patients. **B**. Numbers of detected genes and percentage of differentially expressed genes in UC OM *vs*. CD OM and in UC MES *vs*. CD MES. Details of down- and up-regulated genes are indicated. **C**. Functional annotations of genes differentially expressed in UC OM *vs*. UC MES, in UC OM *vs*. CD OM and in UC MES *vs*. CD MES. GO: Gene Ontology-Biological Process. The first five most significant clusters of up- and down-regulated genes are shown.

In order to avoid contamination by inflammatory cells, gene expression was studied in adipocyte cell fractions from OM and MES from UC and CD patients. Clustering of global gene expression of OM and MES from UC and CD patients is shown in Figure 2A. The gene expression profile of OM adipocytes from the two IBD clustered together whereas MES adipocytes formed separate branches**.**

Two independent differential expression analyses were performed: 1) comparison between OM adipocytes from UC and CD and 2) comparison between MES adipocytes from UC and CD. The proportion of genes differentially expressed ranged between 11 and 14% for each analysis (Figure 2B). Figure 2C shows genes down- and up-regulated in visceral fat depots from UC and CD. Comparison of OM adipocytes derived from the two IBD demonstrated that genes related to metallothioneins and apoptosis pathways were up-regulated and genes linked to inflammation, chemotaxis and angiogenesis were down-regulated in UC compared to CD. Genes linked to bacterial response and inflammation (listed in Table 2) were likewise down-regulated in MES adipocytes from UC compared to CD, whereas genes related to growth factor binding were up-regulated. About half (13/29) of bacterial response genes, down-regulated in MES adipocytes from UC, were also down-regulated in OM adipocytes. 

**Table 2:** List of bacterial response genes in MES and OM adipocytes from UC or CD.

**SYMBOL**	**MES UC/MES CD DiffScore**	**MES UC/MES CD FC**	**OM UC/OM CD DiffScore**	**OM UC/OM CD FC**	**DEFINITION**
***CCL20***	-348.14	0.23	-113.36	0.48	chemokine (C-C motif) ligand 20
***SERPINA1***	-155.94	0.24	-42.26	0.44	serpin peptidase inhibitor, clade A member 1
***IL1B***	-348.14	0.31	-125.97	0.56	interleukin 1, beta
***DEFB1***	-348.14	0.34	-290.68	0.20	defensin, beta 1
***LYZ***	-102.98	0.35	-32.27	0.65	lysozyme
***LBP***	-83.87	0.37	-94.19	0.59	lipopolysaccharide binding protein
***IL27RA***	-20.95	0.39	-32.46	0.40	interleukin 27 receptor, alpha
***TNF***	-348.14	0.47	-181.44	0.42	tumor necrosis factor (TNF superfamily, member 2)
***CD14***	-209.08	0.58	-79.25	0.65	CD14 molecule (CD14), transcript variant 1
***PLA2G2A***	-13.91	0.68	-61.87	0.52	phospholipase A2, group IIA
***CCL5***	-111.47	0.69	-23.34	0.58	chemokine (C-C motif) ligand 5
***HIST1H2BK***	-53.20	0.76	-18.13	0.82	histone cluster 1, H2bk
***TIMP4***	-17.87	0.81	-58.52	0.66	TIMP metallopeptidase inhibitor 4

The Differential score (DiffScore) obtained by Genome Studio software (Illumina) is indicated. A differential score <-13 (i.e. P<0.05) was considered statistically significant. For each gene, the fold change (FC) is indicated.

Comparison of microarray patterns in OM adipocytes from UC with results obtained in healthy controls in a previous study [2], revealed that genes related to apoptosis and inflammatory response were up-regulated while those related to metabolic pathways were down-regulated (Figure S1).

**Figure 3:**
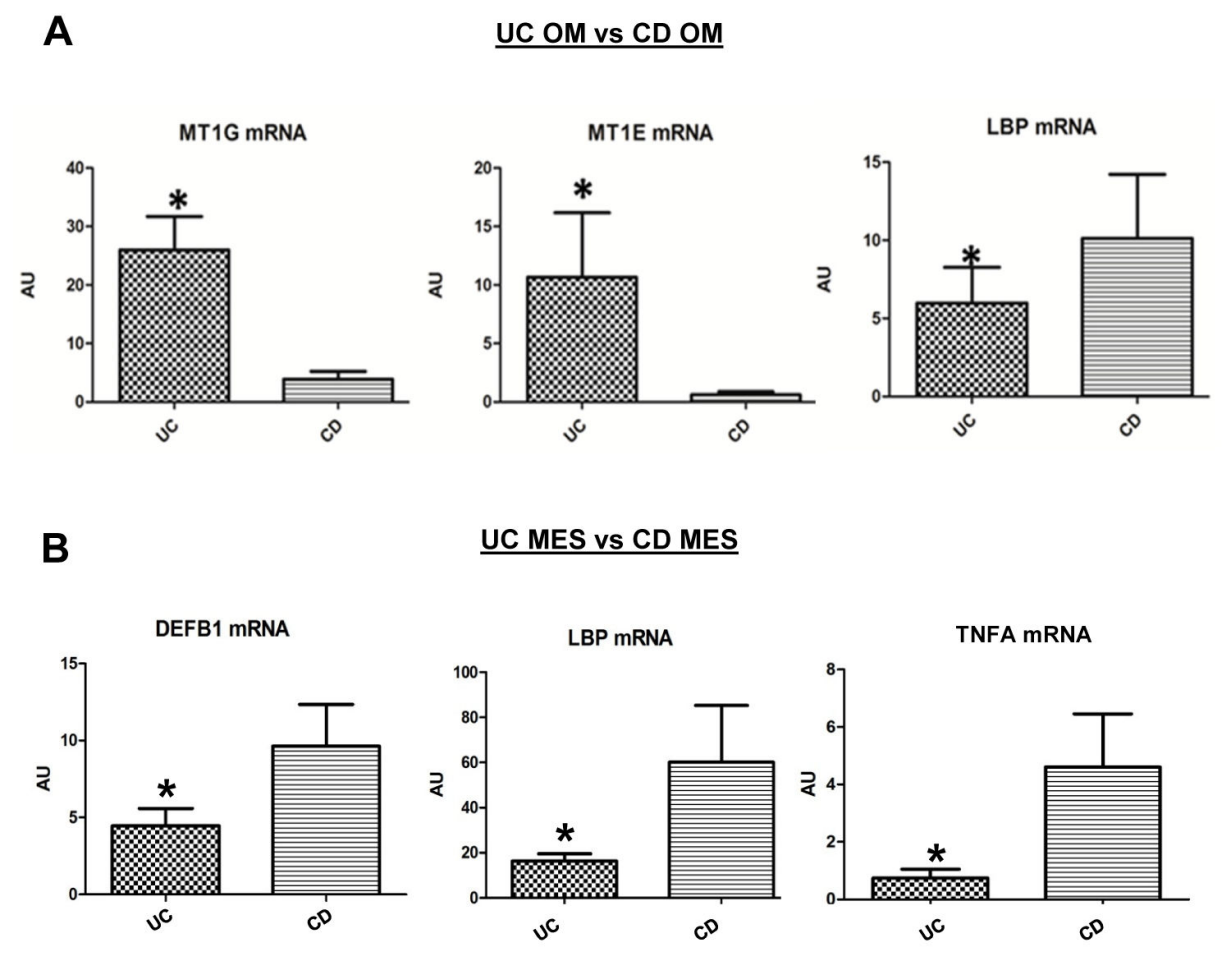
Validation of microarray data by Real Time quantitative PCR. **A**. Expression of metallothionein 1G (MT1G), metallothionein 1E (MT1E), lipopolysaccharide-binding protein (LBP) mRNA in omental adipose tissue (OM) from Crohn's disease (CD) and ulcerative colitis (UC) patients. **B**. Expression of tumor necrosis factor alpha (TNFA), lipopolysaccharide-binding protein (LBP) and defensin beta 1 (DEF1B) in CD and UC MES. Data were normalized to human ribosomal protein LP0 (RPLP0) and are expressed as arbitrary units (AU) ± SE, *P<0.05.

Microarray signals were validated by RTqPCR of selected genes, chosen among those differentially expressed in adipose tissue from UC and CD patients. In agreement with microarray results, *MT1G* and *MT1E* were more expressed and *LBP* less expressed in UC OM than in CD OM (Figure 3A); conversely, *TNFA*, *LBP* and *DEFB1* were less expressed in UC MES than in CD MES (Figure 3B). 

### Detection of intestinal bacteria in visceral fat from IBD patients and effects of bacterial infection on adipocyte proliferation rate

**Figure 4:**
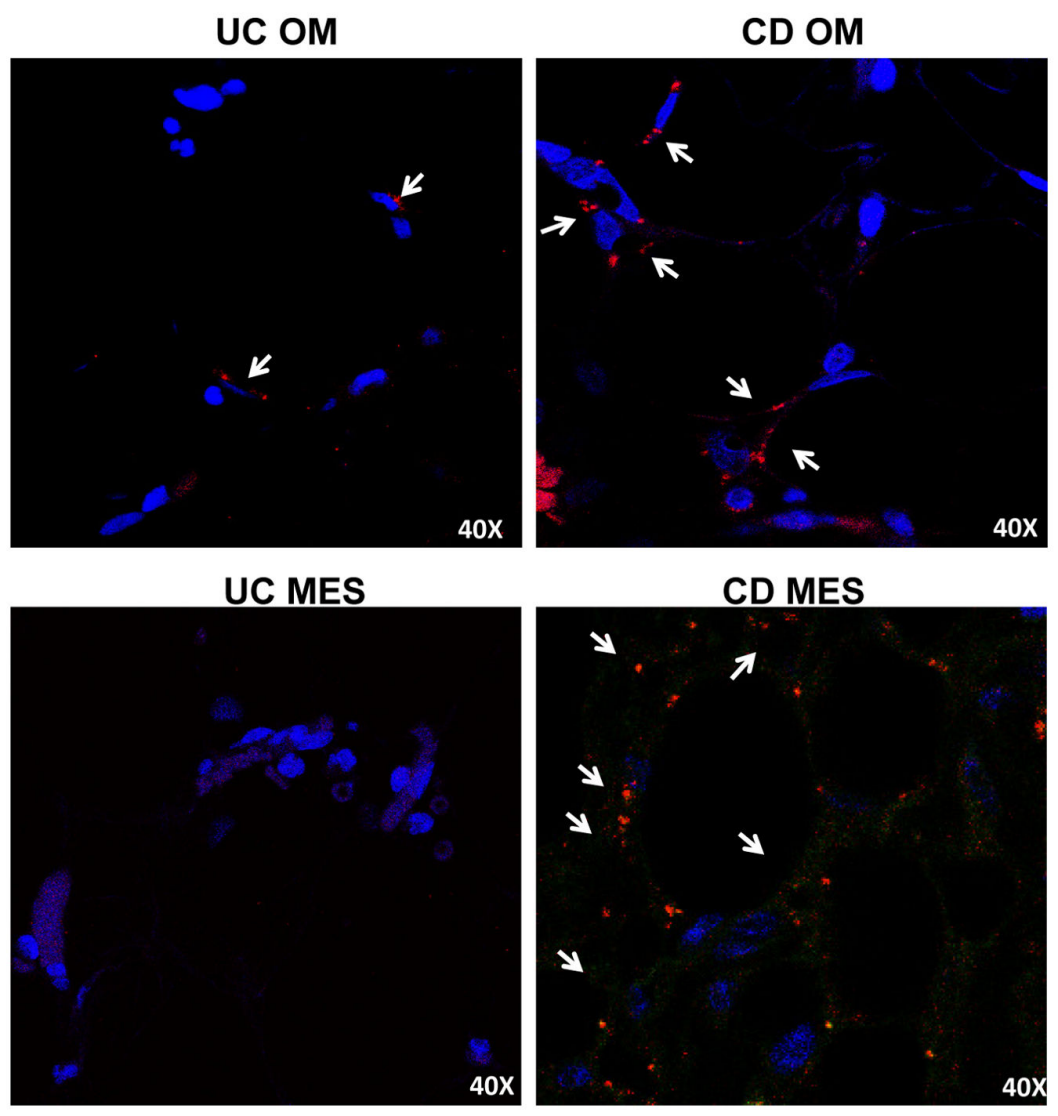
Detection of intestinal bacteria in visceral fat from UC and CD patients. Fluorescent immunodetection of *Enterococcus faecalis* (red) in omental adipose tissue (OM) and mesenteric adipose tissue (MES) from 1 representative ulcerative colitis (UC) and 1 Crohn's disease (CD) patient by confocal microscopy. Magnification is 40X. Nuclei are counterstained in blue (DAPI, 4',6-diamidin-2-fenilindolo).

Confocal microscopy demonstrated a dense perinuclear fluorescent stain for *Enterococcus faecalis* in MES and OM from CD patients, whereas the same adipose tissue depots were only weakly stained in UC specimens (Figure 4).

**Figure 5:**
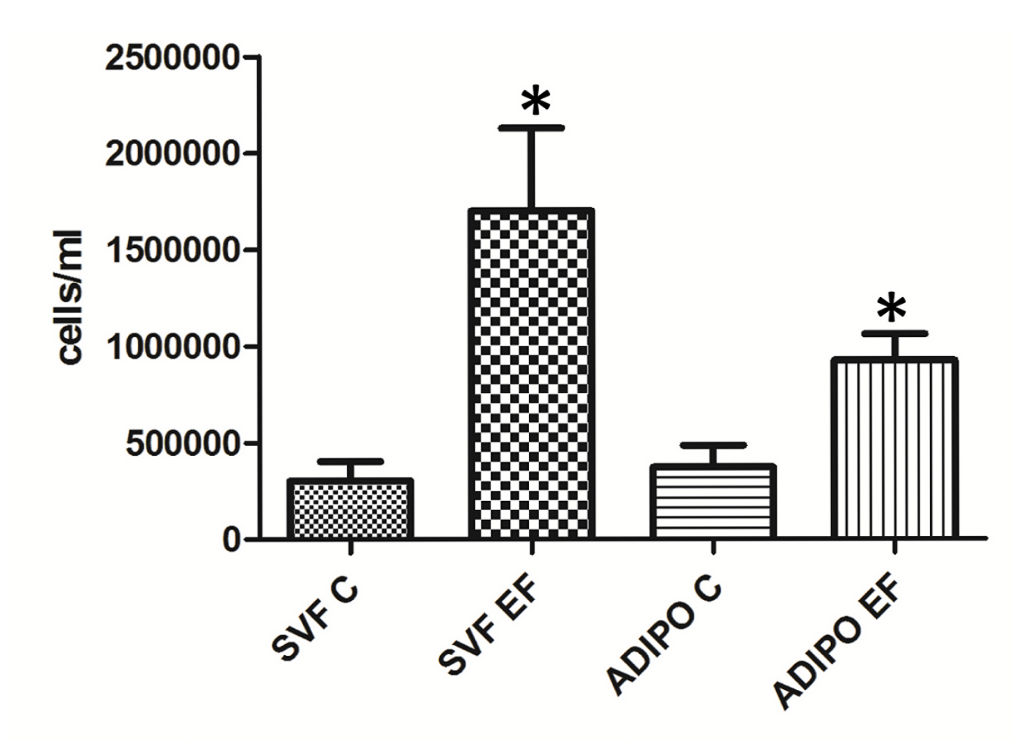
Effects of bacterial infection on adipocyte proliferation rate. Mean cell number ± SE of omental preadipocytes (SVF) and 10-day differentiated adipocytes (ADIPO) from 4 patients (2UC and 2CD) after 24h incubation with (SVF EF and ADIPO EF) or without (SVF C and ADIPO C) 0.001 McFarland *Enterococcus faecalis*. *P<0.05.

Infection of OM preadipocytes with *Enteroccocus faecalis* induced a five-fold increase in proliferation rate compared to non-infected cells (Figure 5). In differentiated adipocytes, infection nevertheless induced a two-fold increase in proliferation rate (Figure 5), accompanied by a 20 % decrease in intracellular lipid content (*data not shown*).

## Discussion

In this study, we provide the first morphological and molecular characterization of visceral fat depots from UC patients. We demonstrate that visceral fat depots from UC patients display less inflammatory features than those from CD patients, both as regards tissue morphology and adipocyte molecular profile. These results are in keeping with the more severe disruption of intestinal barrier, which characterizes CD patients. Visceral adipose tissue inflammation may be driven by intestinal bacteria translocation, possibly proportional to the severity of intestinal lesions. Down-regulation of genes linked to the bacterial response in UC adipocytes tallies with the lower bacterial load in visceral fat from UC compared to CD, whereas up-regulation of genes related to growth factor binding may contribute to the absent fat expansion in UC. The greater bacterial load in visceral fat depots from CD compared to UC might also explain the distinctive development of intra-abdominal hyperplasia in CD. To verify this hypothesis, we infected preadipocytes and differentiated adipocytes with a “non-cytotoxic” load of *Enterococcus faecalis in vitro* and observed an increase in preadipocyte proliferation rate. Further, proliferation was stimulated also in mature adipocytes, which are less prone to proliferate, with the expected concomitant reduction of intracellular lipid content. This finding agrees with the well-known adipocyte plasticity [9] and suggests that mature adipocytes are able to “de-differentiate” (i.e. decrease their intracellular lipid content) and activate proliferative rather than lipogenic pathways during bacterial challenge. In fact, adipocyte cellular differentiation is no longer believed to be irreversible and we herein provide the demonstration that, in conditions such as bacterial load, adipocytes may change their phenotype and reconvert into stromal-vascular cells [9].

Another interesting finding is that bacterial translocation does not target the MES only but may spread to the OM, a depot which is not in direct contact with intestinal lesions. This finding agrees with experimental models of colitis which have shown that gut bacteria translocate from intestinal epithelial cells to mesenteric lymph nodes, blood and peripheral organs including visceral fat [10]. Further, clinical conditions associated with altered gut permeability, e.g. type 2 diabetes, have been associated with increased circulating bacterial DNA both in mice and men [11,12].

A recent study has demonstrated that bacterial translocation to mesenteric lymph nodes and fat is more frequent in patients with CD compared to patients with colorectal cancer, diverticulitis or UC [5]. Our results show for the first time that the extent of bacterial translocation to MES and OM is much greater in CD than in UC. 

The typical perinuclear cellular positivity observed in almost all samples analyzed by confocal microscopy, enabled us to exclude artifacts and cross-contamination during surgical tissue collection and demonstrates that bacteria invade adipocytes in addition to macrophages. Further demonstration that adipocytes may sequester bacteria was obtained in animal and human studies reporting that *Rickettsia prowazekii* and *Mycobacterium tuberculosis* survive in non-replicating state in adipocytes during disease dormancy [13,14]. It may therefore be postulated that adipose tissue acts as a "reservoir" for recrudescent disease caused by dormant infection with enteric commensal bacteria. Within adipocytes, bacteria may be shielded from antibiotics, thus contributing to the variable efficacy of antibiotic therapy in IBD [15].

In conclusion, visceral adipose tissue depots from patients with UC and CD differ significantly in their morphology and molecular profile. The presence of intestinal bacteria in visceral adipocytes and their effect on adipocyte de-differentiation and proliferation may explain adipocyte hyperplasia in creeping fat. The role of bacteria stored into visceral fat on the clinical course of IBD deserves further investigation. 

## Supporting Information

Figure S1 **UP and DOWN-regulated genes in UC versus control omental adipocytes.** Gene Ontology (GO) Biological Process of the first five clusters of up-regulated and down-regulated genes in omental adipocytes (OM) of ulcerative colitis patients (UC) compared to controls. Genes belonging to each category are shown.
(TIF)

## References

[1] Bamias G, Pizarro T, Cominelli F (2011) New Paradigms in the Pathogenesis of IBD. In: Cohen RD, Inflammatory Bowel Disease: Diagnosis and Therapeutics. pp. 41-57.

[2] Zulian A, Cancello R, Micheletto G, Gentilini D, Gilardini L et al. (2012) Visceral adipocytes: old actors in obesity and new protagonists in Crohn's disease? Gut 61: 86-94. doi:10.1136/gutjnl-2012-302514b.33. PubMed: 21930728. 21930728

[3] Goossens GH, Moors CC, van der Zijl NJ, Venteclef N, Alili R et al. (2012) Valsartan improves adipose tissue function in humans with impaired glucose metabolism: a randomized placebo-controlled double-blind trial. PLOS ONE 7: e39930 PubMed: 22768174. 22768174 10.1371/journal.pone.0039930 PMC3386933

[4] Shen J, Obin MS, Zhao L (2013) The gut microbiota, obesity and insulin resistance. Mol Aspects Med 34: 39–58. doi:10.1016/j.mam.2012.11.001. PubMed: 23159341. 23159341

[5] Peyrin-Biroulet L, Gonzalez F, Dubuquoy L, Rousseaux C, Dubuquoy C et al. (2012) Mesenteric fat as a source of C reactive protein and as a target for bacterial translocation in Crohn's disease. Gut 61: 78-85. doi:10.1136/gutjnl-2011-300370. PubMed: 21940721. 21940721 PMC3230831

[6] Huang da W, Sherman BT, Lempicki RA (2009) Systematic and integrative analysis of large gene lists using DAVID bioinformatics resources. Nat Protoc, 4: 44-57. PubMed: 19131956. 19131956 10.1038/nprot.2008.211

[7] Brazma A (2009) Minimum Information About a Microarray Experiment (MIAME)--successes, failures, challenges. Scientific. World J 9: 420-423. doi:10.1100/tsw.2009.57. PMC5823224 19484163

[8] Goossens H (1999) The epidemiology of vancomycin-resistant enterococci. Curr Opin Infect Dis 12: 537–541. doi:10.1097/00001432-199912000-00002. PubMed: 17035818. 17035818

[9] Cinti S (2012) The adipose organ at a glance. Dis Model. J Mech 5: 588-594. 10.1242/dmm.009662 PMC3424455 22915020

[10] Berg RD (1999) Bacterial translocation from the gastrointestinal tract. Adv Exp Med Biol 473: 11-30. doi:10.1007/978-1-4615-4143-1_2. PubMed: 10659341. 10659341

[11] Amar J, Chabo C, Waget A, Klopp P, Vachoux C et al. (2011) Intestinal mucosal adherence and translocation of commensal bacteria at the early onset of type 2 diabetes: molecular mechanisms and probiotic treatment. EMBO. Mol Med 3: 559-572. 10.1002/emmm.201100159 PMC3265717 21735552

[12] Amar J, Serino M, Lange C, Chabo C, Iacovoni J et al. (2011) Involvement of tissue bacteria in the onset of diabetes in humans: evidence for a concept. Diabetologia 54: 3055-3061. doi:10.1007/s00125-011-2329-8. PubMed: 21976140. 21976140

[13] Bechah Y, Paddock CD, Capo C, Mege JL, Raoult D (2010) Adipose tissue serves as a reservoir for recrudescent Rickettsia prowazekii infection in a mouse model. PLOS ONE 5: e8547. doi:10.1371/journal.pone.0008547. PubMed: 20049326. 20049326 PMC2797295

[14] Neyrolles O, Hernández-Pando R, Pietri-Rouxel F, Fornès P, Tailleux L et al. (2006) Is adipose tissue a place for Mycobacterium tuberculosis persistence? PLOS ONE 1: e43. doi:10.1371/journal.pone.0000043. PubMed: 17183672. 17183672 PMC1762355

[15] Khan KJ, Ullman TA, Ford AC, Abreu MT, Abadir A et al. (2011) Antibiotic therapy in inflammatory bowel disease: a systematic review and meta-analysis. Am J Gastroenterol 106: 661-673. doi:10.1038/ajg.2011.72. PubMed: 21407187. 21407187

